# Accuracy of genotype imputation based on random and selected reference sets in purebred and crossbred sheep populations and its effect on accuracy of genomic prediction

**DOI:** 10.1186/s12711-015-0175-8

**Published:** 2015-12-22

**Authors:** Nasir Moghaddar, Klint P. Gore, Hans D. Daetwyler, Ben J. Hayes, Julius H. J. van der Werf

**Affiliations:** Cooperative Research Centre for Sheep Industry Innovation, Armidale, NSW 2351 Australia; School of Environmental and Rural Science, University of New England, Armidale, NSW 2351 Australia; Animal Genetics & Breeding Unit (AGBU), University of New England, Armidale, NSW 2351 Australia; Biosciences Research Division, Department of Economic Development, Jobs, Transport and Resources, Bundoora, VIC Australia; School of Applied Systems Biology, La Trobe University, Bundoora, VIC Australia

## Abstract

**Background:**

The objectives of this study were to investigate the accuracy of genotype imputation from low (12k) to medium (50k Illumina-Ovine) SNP (single nucleotide polymorphism) densities in purebred and crossbred Merino sheep based on a random or selected reference set and to evaluate the impact of using imputed genotypes on accuracy of genomic prediction.

**Methods:**

Imputation validation sets were composed of random purebred or crossbred Merinos, while imputation reference sets were of variable sizes and included random purebred or crossbred Merinos or a group of animals that were selected based on high genetic relatedness to animals in the validation set. The Beagle software program was used for imputation and accuracy of imputation was assessed based on the Pearson correlation coefficient between observed and imputed genotypes. Genomic evaluation was performed based on genomic best linear unbiased prediction and its accuracy was evaluated as the Pearson correlation coefficient between genomic estimated breeding values using either observed (12k/50k) or imputed genotypes with varying levels of imputation accuracy and accurate estimated breeding values based on progeny-tests.

**Results:**

Imputation accuracy increased as the size of the reference set increased. However, accuracy was higher for purebred Merinos that were imputed from other purebred Merinos (on average 0.90 to 0.95 based on 1000 to 3000 animals) than from crossbred Merinos (0.78 to 0.87 based on 1000 to 3000 animals) or from non-Merino purebreds (on average 0.50). The imputation accuracy for crossbred Merinos based on 1000 to 3000 other crossbred Merino ranged from 0.86 to 0.88. Considerably higher imputation accuracy was observed when a selected reference set with a high genetic relationship to target animals was used vs. a random reference set of the same size (0.96 vs. 0.88, respectively). Accuracy of genomic prediction based on 50k genotypes imputed with high accuracy (0.88 to 0.99) decreased only slightly (0.0 to 0.67 % across traits) compared to using observed 50k genotypes. Accuracy of genomic prediction based on observed 12k genotypes was higher than accuracy based on lowly accurate (0.62 to 0.86) imputed 50k genotypes.

## Background

Genomic evaluation refers to prediction of breeding values of selection candidates based on single nucleotide polymorphism (SNP) genotypes that are in linkage disequilibrium (LD) with quantitative trait loci (QTL) and a prediction equation obtained from a group of animals with both phenotypes and genotypes, which is known as the reference population [1]. The reliability of genomic estimated breeding values (GEBV) depends on several factors, such as the size and structure of the reference population and density of genome-wide marker genotypes [2–4]. Denser marker sets are more likely to provide sufficient LD between QTL and SNPs, which can lead to a higher predictive ability and higher accuracy of GEBV [2, 5].

The cost of genotyping increases as more markers are included in the genotyping arrays and this could be a major restriction for large-scale application of genomic evaluation. Instead, low-density SNP arrays are more affordable and can be used directly for genomic evaluation in industry. However, genomic prediction based on low-density SNPs could be more trait-/breed-specific [6] or result in low accuracy of genomic evaluations. A number of studies have compared the effect of SNP density on genomic prediction, mainly from low- to medium-density, based on simulation or real data analyses and have shown a considerable improvement in prediction accuracy by increasing the density of SNP arrays, e.g., [6–10].

Another strategy to achieve higher genomic prediction accuracy from low-density SNP sets is to genotype industry animals with a low-density SNP array and then to infer the un-typed SNP genotypes to a denser marker array based on a reference set via genotype imputation [11, 12]. Genotype imputation refers to statistical inference of un-typed marker genotypes in a set of low-density genotyped animals (imputation test set) based on a group of animals that are genotyped with higher density marker arrays (imputation reference set) [13].

In the Australian sheep industry, GEBV are available via routine genetic evaluations [14]. Moreover, a low-density ovine SNP chip (12k Illumina-Ovine) has been designed for low-cost genotyping of selection candidates to be used in ram breeding flocks. The low-density SNP genotypes can be imputed to 50k SNP density based on available genotypes from a large multi-breed resource flock [15, 16]. This flock consists of purebred Merinos and a large number of crossbred animals, mostly rams from maternal and terminal breeds crossed to Merino ewes. The questions are what imputation accuracies can be achieved when imputing 12 to 50k genotype data and how does that accuracy depend on the size and composition of the reference population. Such information is essential in order to devise the best imputation strategy. Furthermore, the impact of using imputed genotypes on accuracy of genomic evaluations needs to be studied.

The objectives of this study were: (1) to investigate the accuracy of genotype imputation from an evenly spaced low- (Illumina-Ovine 12k) to medium-density (Illumina-Ovine 50k) SNP array in purebred and crossbred Merino sheep populations based on a random or selected imputation reference set and (2) to compare the accuracy of GEBV based on imputed 50k genotypes that are associated with variable imputation accuracies to that of GEBV that are predicted based on observed 50 and 12k SNP genotypes.

## Methods

### Resource flock

Imputation test sets and reference sets were subsets of genotype data selected from a large multi-breed sheep resource flock. The resource flock consisted of purebred and crossbred Merino sheep and was designed as the reference population for genomic prediction studies in Australian sheep breeds. It comprised 22,004 animals genotyped with a 50k SNP density (Illumina-Ovine 50k) and phenotyped for several production traits. The resource flock originated from about 500 sires, such that the animals used in this study belonged to a large number of half-sib families. More information about the resource flock is in Van der Werf et al. [15] and White et al. [17]. The 50k Ovine SNP chip (Illumina Inc., SanDiego, CA, USA) provided 48,599 SNPs for animals in the resource flock after editing the data via genotype quality control. Individual SNP genotype records were removed if the call rate was less than 90 %, the GC (GenCal) score was less than 0.6, the SNP heterozygosity was more than 3 standard deviations away from the mean, the SNP minor allele frequency was less than 0.01, the SNP was located on chromosome X or Y, and if SNP genotypes deviated greatly from Hardy–Weinberg equilibrium (P < 1 × 10^−15^). The entire genotype record was also removed if the correlation with genotypes of another sample was more than 0.98. Following quality control, the sporadic missing genotypes in all resource data (up to 10 %) were imputed using Beagle software v3.2 [18]. The 12k SNP array provided 12,468 SNPs, which was reduced to 11,377 SNPs after removing un-mapped SNPs and performing quality control as described above. The final 12k SNP panel was used to extract 12k genotypes for animals in the test set by masking the remaining SNP genotypes of the 50k SNP array.

### Imputation test sets and reference sets

**Fig. 1:**
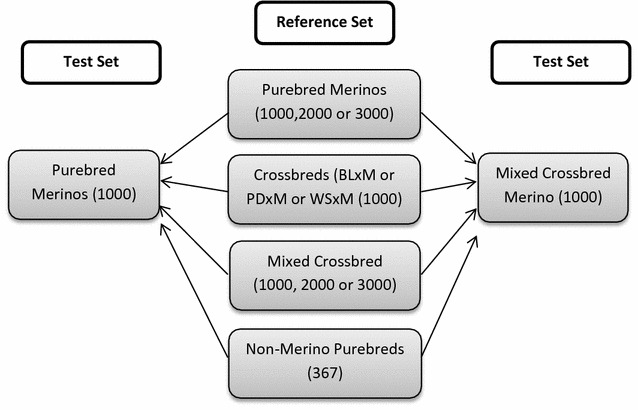
Overview of imputation test sets (validation animals) and random reference sets. *Numbers* in parenthesis are the size of each population. *BL* Border Leicester, *M* Merino, *PD* Poll Dorset, *WS* White Suffolk

Imputation test sets (target animals) consisted of 1000 purebred Merinos, 1000 mixed crossbred Merinos, or 500 crossbred Merinos (BLxM or PDxM or WSxM). Random reference sets consisted of 1000, 2000 or 3000 purebred Merinos; 1000 crossbred Merinos; 1000, 2000 and 3000 mixed crossbred Merinos (combination of BLxM, PDxM and WSxM) or 367 available non-Merino purebreds (purebred BL, PD and WS) extracted from the large multi-breed sheep resource flock. Figure 1 is an overview of the imputation scenarios from the random reference sets.

In addition to imputing from a random reference set, we also tried to impute from a reference set that was chosen to be informative for all animals in the imputation test set. This selected reference set had the same size as the random reference set (2000 animals) and was based on calculating first the genomic relationship between animals in the test set and all reference animals (i.e. the multi-breed resource flock) based on common 12k genotypes and VanRaden’s algorithm [19]. In the next step, for each animal in the test set, the 20 most related animals were selected from the reference set. The final selected reference set included all 20 animals selected for each animal in the test set after removing duplicate animals. If the resulting set included less than 2000 animals, we increased the number of selected animals per animal in the test set from 20 to 21 or more if required. If the algorithm led to more than 2000 animals, animals that had the lowest average relationship to all test set animals were deleted from the final reference set.

### Imputation program software and imputation accuracy

The Beagle software program v3.2 [18] was used to impute un-typed genotypes in the test set. Imputation was performed separately for each chromosome and was based on 10 iterations. The accuracy of imputation was calculated for each individual in the test set as the Pearson correlation coefficient between observed and imputed 50k genotypes, after discarding the 12k observed genotypes. If imputation was based on a selected reference set, imputation accuracies were also based on the Pearson correlation coefficient of each imputed SNP across test individuals, as well as on the Pearson correlation coefficient between imputed and observed genotypes for each test individual.

### Genomic prediction

The effect of using imputed genotypes on accuracy of genomic prediction was assessed in purebred Merinos. For this, genomic best linear unbiased prediction (GBLUP) was performed based on 1000 purebred Merino as the genomic prediction reference population (which was also used as imputation test set (see Fig. 1)). The genomic relationship matrix (**G**) was calculated based on VanRaden’s algorithm [19] using 50 or 12k observed genotypes or 50k imputed genotypes associated with high or low imputation accuracies. ASReml program software [20] was used to obtain GEBV based on the following linear mixed model:

$${\mathbf{y}} = {\mathbf{Xb}} + {\mathbf{Zg}} + {\mathbf{Ww}} + {\mathbf{Z}}_{ 1} {\mathbf{Qq}} + {\mathbf{e}}.$$

In this model, **y** is a vector of phenotypes, **b** is a vector of fixed effects, **g** is a vector of random additive genetic effects, **w** is a vector of random maternal effects, **q** is a vector of breed effects, and **X**, **Z**, **W** and **Z**_**1**_ are incidence matrices relating the former effects to phenotypes. **Q** is a matrix with breed proportions (including Merino strains) for each animal and **e** is a vector of random residuals. Vectors **g**, **e,w** and **q** were assumed normally distributed as: ${\mathbf{g}} \sim N\left( {0,\mathbf{G}\delta_{g}^{2} } \right)$, ${\mathbf{e}} \sim N\left( {0,\mathbf{I}\delta_{e}^{2} } \right), {\mathbf{w}} \sim N\left( {0,\mathbf{I}\delta_{w}^{2} } \right)$ and ${\mathbf{q}} \sim N\left( {0,\mathbf{I}\delta_{q}^{2} } \right)$. The fixed effects included in the model were birth type, rearing type, gender, age at measurement, weight at measurement and contemporary group, which was a combination of flock, birth year and management group effects. Accuracy of GEBV was assessed on a group of validation sires based on the Pearson correlation between GEBV and accurate breeding value calculated based on pedigree and phenotypes known as Australian sheep breeding values (ASBV). ASBV resulted from the national genetic evaluation system by excluding any data from the genomic prediction reference population. The validation population consisted of 175 older Merino sires that each had a substantial number of progeny recorded, with an ASBV accuracy that ranged from 0.70 to 0.99 (on average 0.88).

## Results

### Imputation accuracy in purebred Merinos

#### Imputation accuracy in purebred Merinos based on other purebred Merinos

**Fig. 2:**
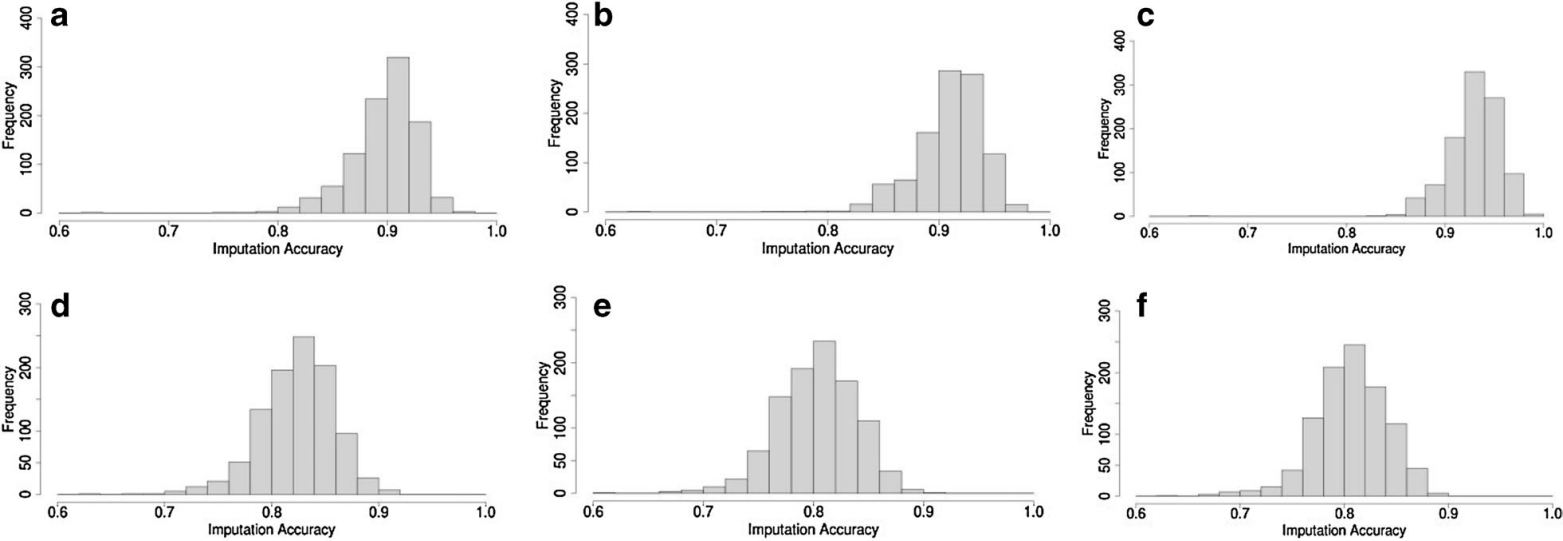
Distribution of imputation accuracies for 1000 purebred Merinos based on 1000 (**a**), 2000 (**b**) and 3000 (**c**) purebred Merinos or based on 1000 BLxM (**d**), 1000 PDxM (**e**) and 1000 WSxM crossbred Merino (**f**). *BL* Border Leicester, *M* Merino, *PD* Poll Dorset, *WS* White Suffolk

The distribution of imputation accuracy of 1000 purebred Merinos based on a random set of 1000, 2000 or 3000 other purebred Merinos in the reference set is in Fig. 2a–c. A relatively high average imputation accuracy was observed with a wide range of values. A significant improvement in accuracy was observed by increasing the size of the reference set. The average imputation accuracy based on 1000 purebred Merino was equal to 0.91 and increased from 0.93 and 0.96 based on 2000 and 3000 purebred Merinos, respectively. The results also show that a larger reference set leads to a smaller range of imputation accuracies. The relatively wide range of imputation accuracies, in particular when based on the smaller reference sets, is due to the genetic variability of the animals in the test set and to the random reference set not expected to be informative for imputation across all test set animals. The average, standard deviation and range of genomic relatedness between animals of a random purebred reference set and animals of a purebred Merino test set were 0.00, 0.02 and −0.07 to 0.37, respectively, across the three purebred reference sets.

#### Imputation accuracy in purebred Merinos based on crossbred Merinos

Imputation accuracies of 1000 purebred Merinos based on 1000 crossbred Merinos (BLxM, PDxM or WSxM) are in Fig. 2d–f. As expected, imputation accuracy based on crossbreds was lower than imputation based on purebred Merinos. The average imputation accuracy was equal to 0.82 and imputation accuracies ranged from 0.70 to 0.92. The results showed almost no difference in imputation accuracy of purebred Merinos across the three Merino crossbred reference sets (Fig. 2d–f). Both the lower accuracy and the lack of notable difference in imputation accuracies between different crossbred Merino reference sets (BLxM, PDxM or WSxM) suggest that the non-Merino breed haplotypes (BL, PD or WS) were not informative for the imputation of Merino breed haplotypes.

#### Imputation accuracy in purebred Merinos based on mixed crossbred Merinos

**Fig. 3:**
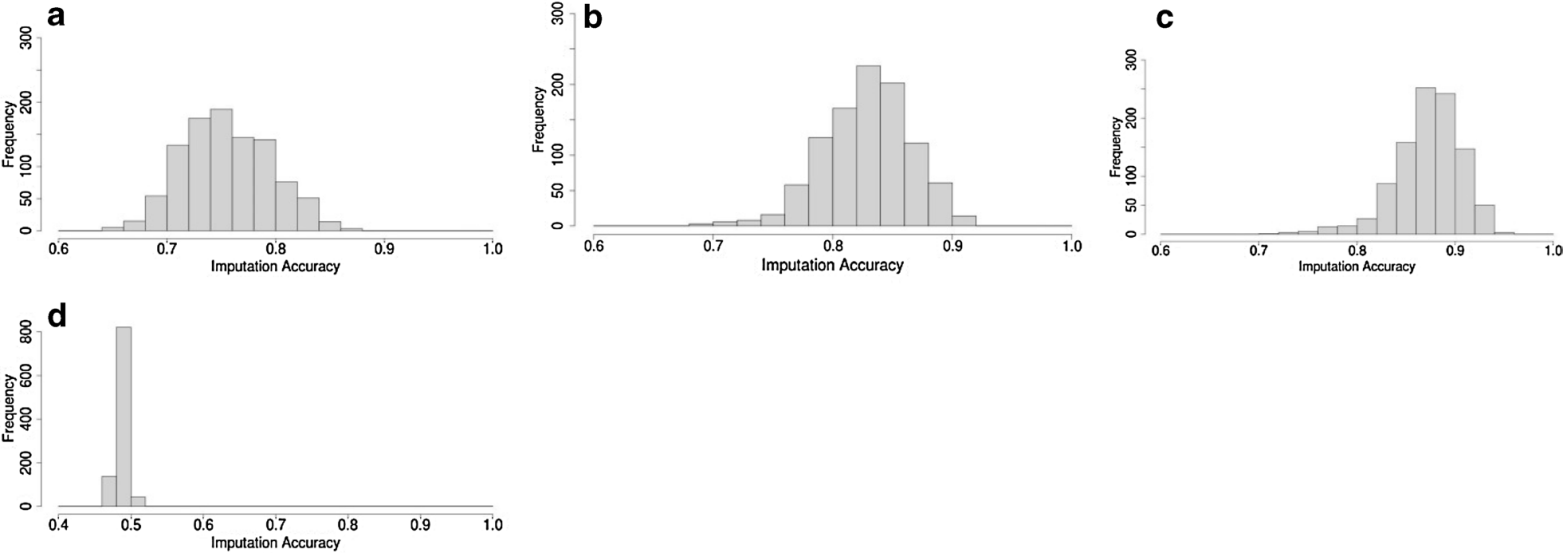
Distribution of imputation accuracies for 1000 purebred Merinos based on 1000 (**a**), 2000 (**b**), 3000 (**c**) mixed crossbred Merinos and based on 367 non-Merino purebreds (**d**)

Figure 3a–c show the distribution of imputation accuracies of purebred Merinos based on mixed crossbred Merinos (BLxM, PDxM and WSxM equally represented). The average accuracy was equal to 0.76, 0.84 and 0.88 based on 1000, 2000 and 3000 mixed crossbred Merinos, respectively, which was considerably lower than imputation from purebred Merinos. The range of imputation accuracies based on crossbreds was also much larger compared to imputation based on purebred Merinos. Moreover, the comparison of Fig. 3a with Fig. 2d, e or f shows no considerable difference in imputation accuracy for purebred Merinos based on 1000 crossbred Merino or using 1000 mixed crossbred Merinos. Note that in these four cases, the crossbred reference populations provided a similar number of informative Merino haplotypes.

#### Imputation accuracy of purebred Merinos based on non-Merino purebred animals

Figure 3d shows the distribution of imputation accuracies of purebred Merinos from the 367 available animals of the non-Merino purebred reference set (mixture of purebreds BL, PD and WS). The results indicated very low imputation accuracy from across-breed genotypes. The average imputation accuracy was equal to 0.50 and values ranged from 0.46 to 0.60.

### Imputation accuracy in mixed crossbred Merinos

#### Imputation accuracy in mixed crossbred Merinos based on other mixed crossbred Merinos

**Fig. 4:**
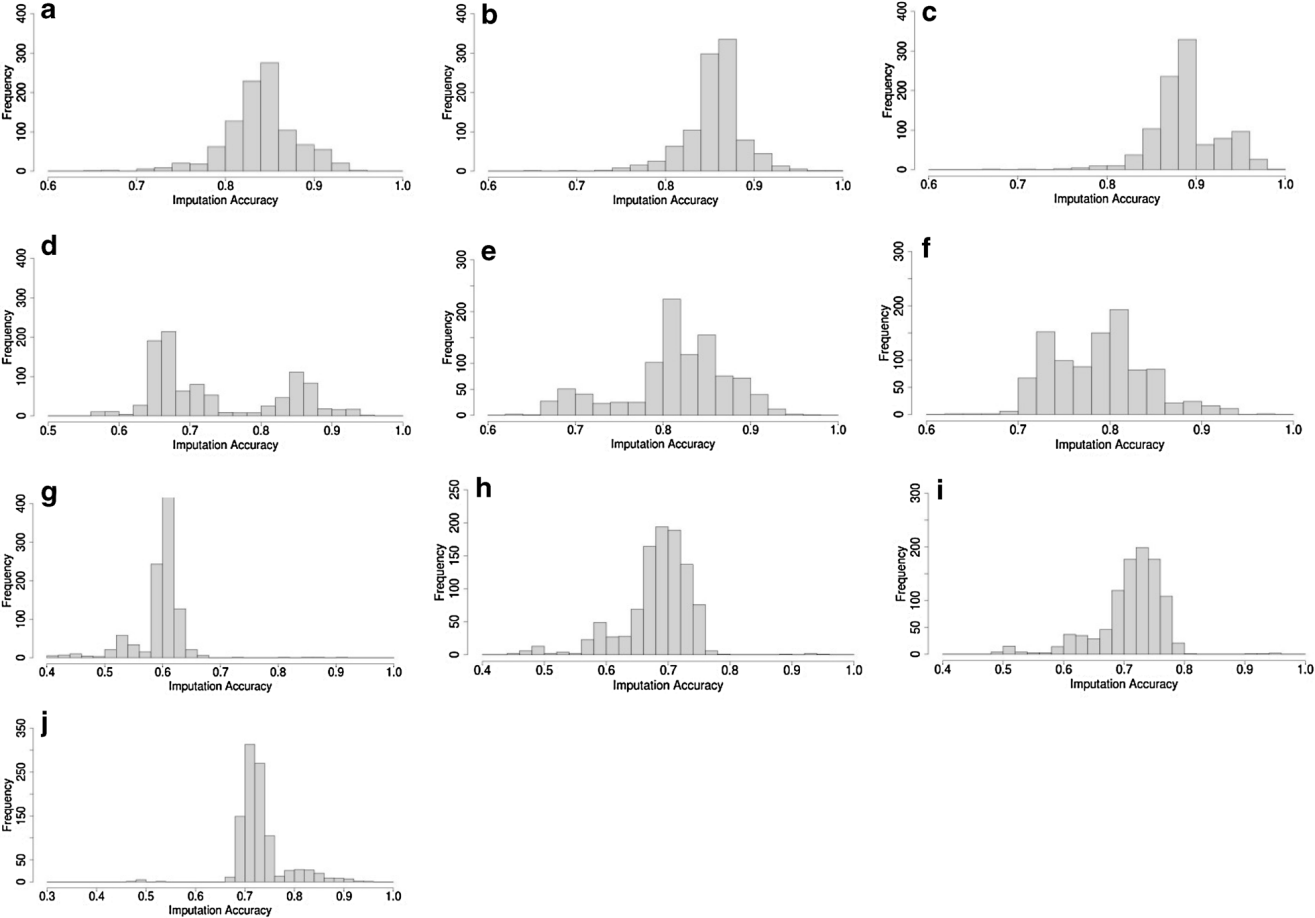
Distribution of imputation accuracies for 1000 mixed crossbred Merinos based on 1000 (**a**), 2000 (**b**), 3000 (**c**) mixed crossbred Merinos, based on 1000 different single crossbred Merinos [BLxM (**d**), PDxM (**e**) and WSxM (**f**)], based on 1000 (**g**), 2000 (**h**) and 3000 (**i**) purebred Merino or based on 367 non-Merino purebreds (**j**). *L* Border Leicester, *M* Merino, *PD* Poll Dorset, *WS* White Suffolk

Figure 4a–c show the distribution of imputation accuracies in mixed crossbred Merinos (mixed BLxM, PDxM and WSxM) based on 1000, 2000 and 3000 random mixed crossbred Merinos. Average imputation accuracies were equal to 0.86, 0.88 and 0.90 based on 1000, 2000 and 3000 mixed crossbred, respectively, and the overall range of the accuracies was 0.68 to 0.98. Similar to imputation in purebred Merinos, imputation accuracy increased when using a larger reference set. Comparison of Fig. 4a–c with Fig. 2d–f showed that imputation accuracy of crossbred Merinos from other crossbred Merinos was higher than that of purebred Merinos from a crossbred Merinos reference set. This could be explained by the fact that the crossbred Merino reference set provided haplotypes that were common to both parental breeds of the crossbred test set.

#### Imputation accuracy of mixed crossbred Merinos from crossbred Merinos

The distribution of imputation accuracies of 1000 mixed crossbred Merinos based on 1000 random BLxM or PDxM or WSxM crossbred reference sets are in Fig. 4d–f. Compared to imputation from mixed crossbreds sets of the same size (Fig. 4a), the average imputation accuracy was lower and the range of accuracies was also considerably larger. This is because a mixed crossbred Merino test set has fewer haplotypes in common with a crossbred Merino reference set (BLxM or PDxM or WSxM) than with a mixed crossbred Merino reference set. The mixed crossbred Merino reference set has haplotypes from Merino, BL, PD and WS breeds, while each crossbred Merino reference set covers only haplotypes from either Merino and BL (Fig. 4d), Merino and PD (Fig. 4e), or Merino and WS breeds (Fig. 4f). Note that the distribution of imputation accuracies was wider and tended towards a bimodal distribution (Fig. 4d–f).

#### Imputation accuracy of mixed crossbred Merinos from purebred Merinos or from non-Merino purebreds

The distribution of imputation accuracies of crossbred Merinos using a reference set of 1000, 2000 and 3000 purebred Merinos are in Fig. 4g–i, respectively. Imputation accuracy was low and ranged from 0.43 to 0.72. A larger purebred Merino reference set (2000 or 3000) provided higher average imputation accuracy but the range of accuracies was still very large (0.48 to 0.80).

Imputation accuracy of mixed crossbred Merinos based on a reference set of non-Merino purebred animals was also low and on average equal to 0.76 (Fig. 4j). Note that imputation accuracy in this case was higher than that of purebred Merinos from non-Merino purebreds (Fig. 3d). This is because breed haplotypes in the non-Merino purebred reference set, which are in common with the first-cross Merino test sets (BL, PD and WS haplotypes) are of paternal origin and, therefore are likely to be more similar than the shared Merino haplotypes that are of maternal origin.

### Imputation accuracy based on a selected reference set

**Fig. 5:**
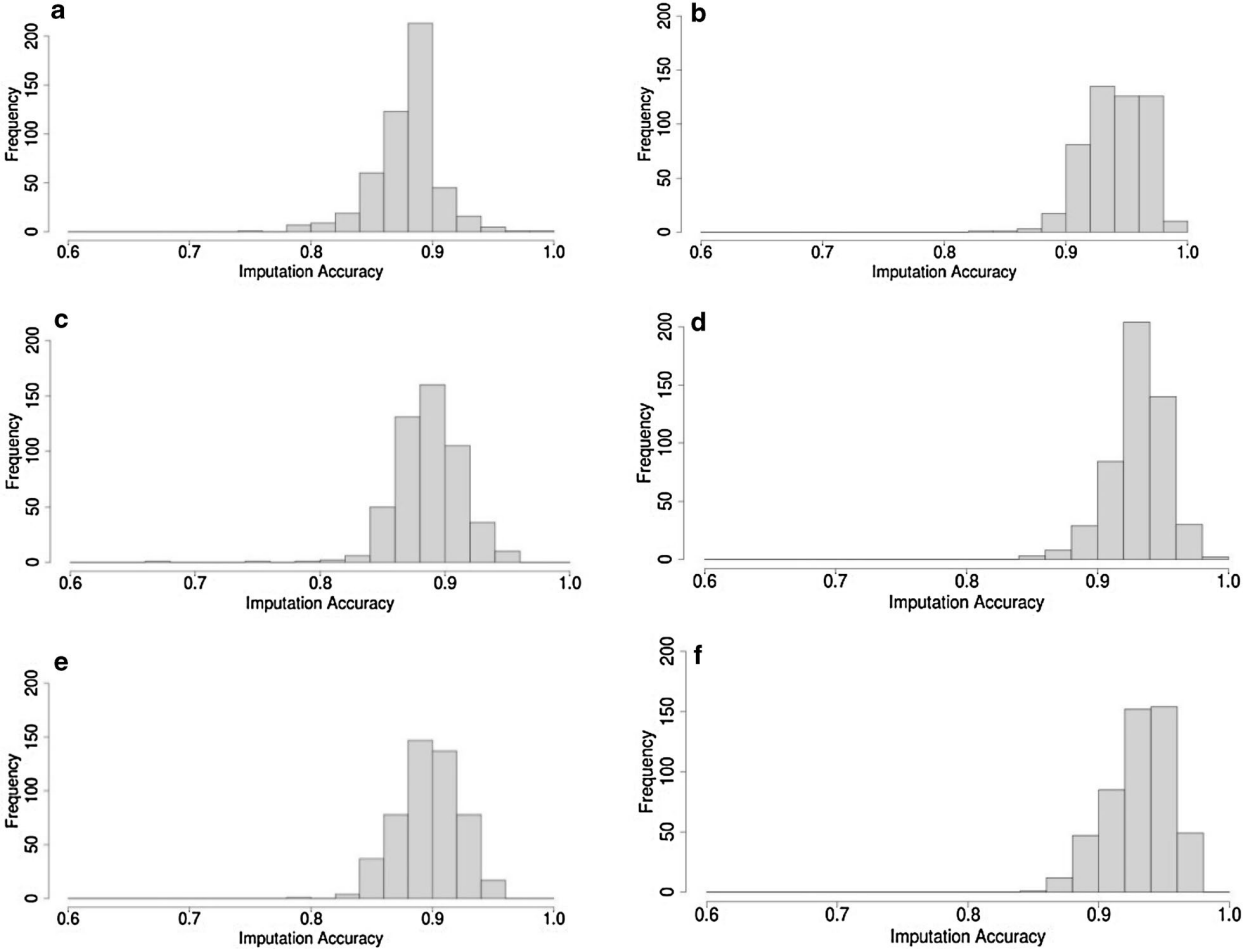
Distribution of imputation accuracies of 1000 BLxM crossbreds based on 2000 random crossbreds (**a**) or 2000 selected reference set (**b**), for 1000 PDxM crossbreds based on 2000 random crossbreds (**c**) or 2000 selected reference set (**d**) and for 1000 WSxM crossbreds based on 2000 random crossbreds (**e**) or 2000 selected reference set (**f**). *L* Border Leicester, *M* Merino, *PD* Poll Dorset, *WS* White Suffolk

Figure 5 compares the distribution of imputation accuracies for test sets of 500 BLxM, 500 PDxM or 500 WSxM crossbred Merinos based on a reference set of 2000 random crossbreds with the accuracy estimated from the 2000 selected reference set in which all animals had high genetic relatedness to all the animals in the test set. For all three test sets, results showed a significant increase in average imputation accuracy when a selected reference set was used and also a significant decrease in the range of accuracies. The average imputation accuracy for crossbred Merinos based on a random reference set of 2000 crossbreds was equal to 0.88. This increased from 0.96 to 0.97 when using the 2000 selected reference set. The range of imputation accuracies based on a selected reference set was also smaller (0.88 to 1.00) compared to the random crossbred reference set (0.76 to 0.95). These results show that the size of the reference set is more important when genomic relationships between imputation test set and reference set animals are lower.

**Fig. 6:**
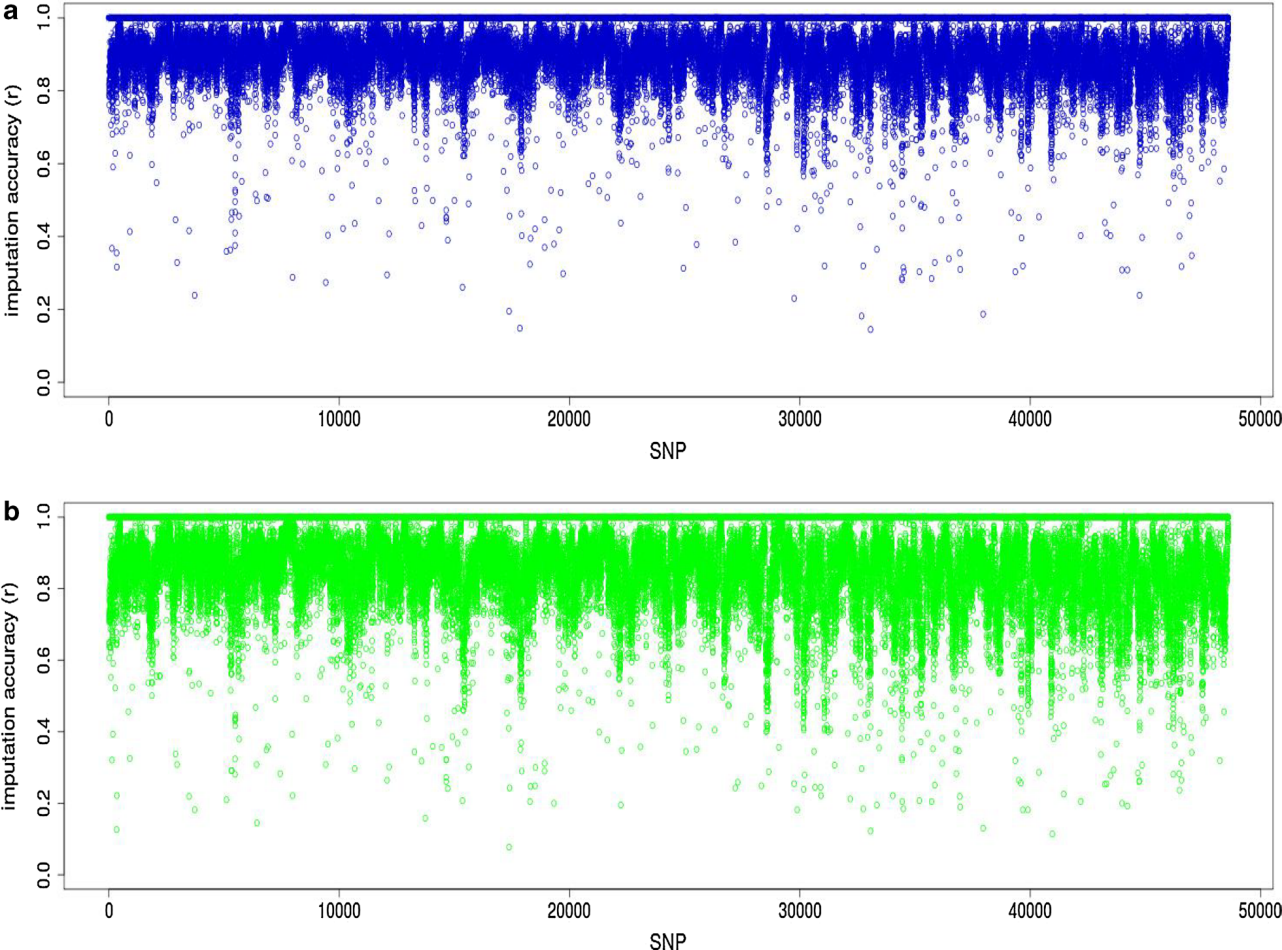
SNP imputation accuracies for 500 BLxM crossbreds from 2000 selected (**a**) vs. 2000 random crossbred reference set (**b**). *L* Border Leicester, *M* Merino

Figure 6 shows the imputation accuracy of individual SNPs based on a selected vs. random reference set. Imputation accuracy of individual SNP genotypes was significantly higher when it was based on the selected than on the random reference set. The average imputation accuracy of individual SNPs increased from 0.77 based on a random reference set to 0.87 based on the selected reference set.

### Genomic prediction based on imputed genotypes

Table 1 shows the accuracy of genomic prediction for three different Merino sheep production traits (post-weaning weight (PWWT), scanned eye muscle depth (EMD) and yearling greasy fleece weight (YGFW)) using observed 50 or 12k genotypes vs. using imputed 50k genotypes with variable imputation accuracies (from 0.55 to 0.60 and from 0.88 to 0.99). The high or low imputation accuracy was related to imputation of 1000 purebred Merinos (used here as the genomic prediction reference population) based on the purebred Merinos, crossbred Merinos or non-Merinos purebred imputation reference sets that were described in the above section on imputation accuracies of purebred Merinos.

**Table 1:** Accuracy of genomic prediction based on observed genotypes (50 or 12k) and imputed 50k genotypes with different accuracies for post-weaning weight (PWW), post-weaning eye muscle depth (PW_EMD) and yearling greasy fleece weight (YGFW) in Merino sheep

Genotypes	Imputation accuracy	PWW	PW_EMD	YGFW
Observed 50k	NA^a^	0.446	0.219	0.585
Imputed 50k − 1^b^	0.88–0.99	0.443	0.219	0.584
Imputed 50k − 2(1)^c^	0.73–0.96	0.428	0.217	0.583
Imputed 50k − 2(2)^d^	0.73–0.96	0.430	0.215	0.582
Imputed 50k − 3^e^	0.62–0.86	0.394	0.184	0.572
Imputed 50k − 4^f^	0.48–0.60	0.381	0.171	0.534
Observed 12k	NA	0.412	0.205	0.552

^a^Not applicable

^b^Imputed from 3000 purebred Merino

^c^Imputed from 3000 crossbred Merino

^d^Imputed from 1000 crossbred Merino and edited for individual SNPs with low imputation accuracy (r < 0.7)

^e^Imputed from 1000 mixed crossbred Merino

^f^Imputed from 367 non-Merino purebreds

The accuracy of genomic prediction based on highly accurate imputed genotypes (ranging from 0.88 to 0.99) was the same or slightly lower than the accuracy from observed 50k genotypes. Accuracy of genomic prediction based on imputed 50k genotypes but with relatively moderate to high accuracies (ranging from 0.73 to 0.96, with an average of 0.885), was up to 4.0 % less than that using observed 50k genotypes, but it was still higher than the accuracy based on observed 12k genotypes. Removing SNPs with low (<0.70) individual imputation accuracies did not increase the genomic prediction accuracy.

The accuracy of genomic prediction decreased by 15.9 to 21.9 % across the three traits when it was based on imputed genotypes with very low accuracy (on average equal to 0.68 and 0.57, respectively). The GEBV accuracy based on 12k genotypes was higher than that based on imputed 50k genotypes with low accuracy.

Table 2 shows the correlation between GEBV based on observed or imputed genotypes, using PWWT as an example. The correlation was high between observed 50k and imputed 50k genotypes with high accuracies but was lower between observed 50k genotypes and imputed genotypes with lower accuracies. The changes in correlation coefficient between GEBV that were estimated based on observed vs. imputed genotypes were similar to the trends observed for the accuracy of GEBV. This correlation pattern between GEBV for PWWT was very similar for the two other traits. Table 3 presents the correlations between the genomic relationship matrices (GRM) based on observed vs. imputed genotypes for animals in the reference population. Correlations between the different GRM followed the same pattern as correlations between GEBV and were higher when the 50k genotypes were imputed more accurately.

**Table 2:** Correlations between genomic estimated breeding values based on observed and imputed genotypes with different accuracies for post weaning weight

Genotypes	Observed 50k	Imputed 50k − 1	Imputed 50k − 2	Imputed 50k − 3	Imputed 50k − 4	Observed 12k
Observed 50k	1.000					
Imputed 50k − 1	0.991	1.000				
Imputed 50k − 2	0.970	0.968	1.000			
Imputed 50k − 3	0.957	0.953	0.957	1.000		
Imputed 50k − 4	0.814	0.812	0.821	0.819	1.000	
Observed 12k	0.915	0.911	0.926	0.916	0.869	1.000

50k − 1: imputation accuracy between 0.88 and 0.99

50k − 2: imputation accuracy between 0.73 and 0.96

50k − 3: imputation accuracy between 0.62 and 0.86

50k − 4: imputation accuracy between 0.55 and 0.60

**Table 3:** Correlations between genomic relationships based on observed and imputed genotypes with different accuracies

Genotypes	Observed 50k	Imputed 50k − 1	Imputed 50k − 2	Imputed 50k – 3	Imputed 50k − 4	Observed 12k
Observed 5k	1.000					
Imputed 50k − 1	0.999	1.000				
Imputed 50k − 2	0.997	0.996	1.000			
Imputed 50k − 3	0.994	0.992	0.995	1.000		
Imputed 50k − 4	0.828	0.825	0.830	0.850	1.000	
Observed 12k	0.992	0.990	0.989	0.987	0.829	1.000

50k − 1: imputation accuracy between 0.88 and 0.99

50k − 2: imputation accuracy between 0.73 and 0.96

50k − 3: imputation accuracy between 0.62 and 0.86

50k − 4: imputation accuracy between 0.55 and 0.60

## Discussion

This study investigated the accuracy of genotype imputation from a commercially available low- (12k) to a medium- (50k) density SNP panel in purebred and crossbred Merino sheep with different strategies for selecting the reference set. Then, accuracies of genomic prediction based on imputed 50k genotypes that had different accuracies, were compared with those based on observed 50 and 12k genotypes. The study was motivated by the need to implement imputation from low-density marker panels into routine genomic evaluation of Australian sheep, which comprises multiple breeds and crossbreds. The results showed higher imputation accuracy for larger reference sets, but a large improvement in accuracy was observed when animals in the reference set were selected to be genetically more related to the target animals. This leads to the general observation that imputation accuracy is driven by the number of relevant haplotypes in the reference population, and for more accurate imputation of crossbred animals, the imputation reference set should have a sufficient number of haplotypes for all the breeds involved in crossbred animals. In fact, these rules are equally relevant when constructing a reference set for genomic prediction, for which accuracy is also related to the number of relevant haplotypes used and their relatedness with the predicted individual.

### Imputation

Larger and more related reference sets provide a greater chance of finding more informative haplotypes for inferring un-typed genotypes of animals in the test set. The additional accuracy that was obtained from larger random reference sets was greater when imputing crossbred animals because crossbreds are genetically more heterogeneous. Increases in imputation accuracy from larger reference sets have been reported for simulation or real data analyses, e.g., [12, 21, 22, 23, 24, 25] but none of these studies explicitly compared imputation of purebreds vs. crossbreds or investigated the effect of selecting the best reference population.

**Fig. 7:**
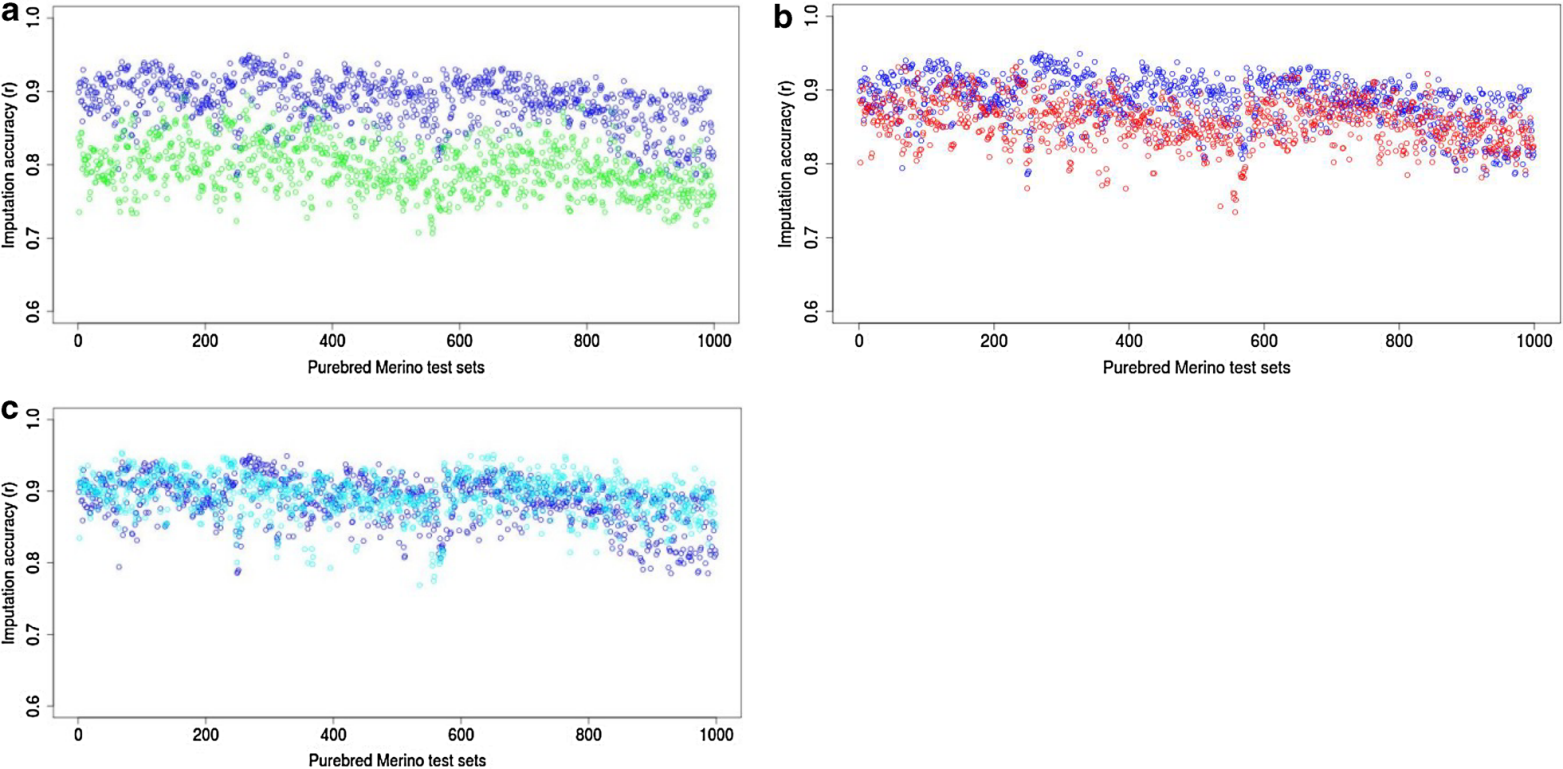
Imputation accuracies of purebred Merinos from 1000 random purebred Merinos (*dark blue*) vs. **a** 1000 crossbred Merinos (*green*), **b** 2000 crossbred Merinos (*red*) and **c** 3000 crossbred Merinos (*light blue*)

The results showed higher imputation accuracy for purebred Merinos when based on a purebred reference set compared to a reference set based on crossbred Merinos or other breeds. While both purebreds and crossbreds can provide informative breed haplotypes for imputation of a purebred target animal, the higher accuracy that was obtained based on purebreds can be explained by the larger number of informative haplotypes. A reference set of 1000 purebred Merinos provides a larger number of (up to 2000) informative Merino breed haplotypes, while a 1000 first-cross Merino reference set provides only up to 1000 informative Merino breed haplotypes. In our study, we observed similar imputation accuracies when using 1000 purebred Merinos or 3000 crossbred Merinos (Fig. 7). The theoretical expectation would be that 2000 random crossbred Merinos provide similar imputation accuracies as a 1000 purebred Merino reference set. However, in our study the value of paternal haplotypes was generally higher than that of maternal haplotypes because the paternal haplotypes were more similar to haplotypes in the test set population. The reason is that the research sheep flocks were genetically connected to each other through the use of common sires via artificial insemination for almost 50 % of the males in the mating program. Maternal haplotypes originated from founding ewes in the research flocks and were genetically more distant from the industry sires that were used across all flocks. Therefore, imputation accuracy not only depends on the number of breed-relevant haplotypes used, but also on the genetic distance between the haplotypes in the reference set and the test set.

We also found that imputation of mixed crossbred Merinos from other mixed crossbred Merinos provided higher accuracies than imputation from crossbreds or from purebred Merinos. The reason is again that the mixed crossbred Merinos set includes more haplotypes relevant to the mixed crossbred reference set; the mixed crossbred reference set contained haplotypes from all four breeds (Merino, BL, PD and WS) that are relevant to the target test set, while crossbred Merinos contain only haplotypes from two breeds.

Smaller or less related reference sets showed a wider range of imputation accuracies, which is in line with previously reported results [12]. This shows that more individuals are poorly imputed based on such reference sets, likely because their haplotypes are not represented in the reference population.

The relevance of a genetically-related reference set to the animals in the test set was clearly shown when we selected animals for a reference set based on their high genomic relatedness to the animals in the test set, which resulted in a very significant improvement in imputation accuracy compared to using a random reference set of the same size. The impact of genetic relatedness between the reference and test animals (such as having direct relatives with the target animals in the reference population) on the accuracy of genotype imputation was previously reported, e.g. [21–23]; however, our results show that selection of the imputation reference set based on relationship is a more general and efficient way to achieve high imputation accuracy. Investigation of the breed component structure of the selected reference sets compared to the random reference sets showed that the proportion of haplotypes of the target breed was considerably larger in the selected reference set than in the random reference set. For instance, the proportion of BL haplotypes in a reference set selected for imputation of crossbred BLxM animals increased from 9.7 to 41.8 % or the proportion of PD haplotypes in a selected reference set for imputation of crossbred PDxM increased from 12.2 to 51.1 %. When selecting a reference set, we also attempted to maintain a high level of diversity among the selected animals by minimizing relationships among them. However, it turned out that this selection criterion had little effect on the animals that were selected because of the relatively strong half-sib family structure of the data.

Imputation processing time increases exponentially with the size of the reference set [18] and this could make the imputation computationally prohibitive. However, our results show that imputation based on a selected reference set can be performed efficiently with high accuracy if sufficient data is available, which should be useful for routine practical genomic evaluations.

We applied a population-based imputation method. Imputation accuracy can potentially be increased by combining population- and family-based imputation. However, the additional accuracy obtained by adding family information is expected to be small, particularly if the reference set is large, because a population-based imputation indirectly exploits family information [18]. Larmer et al. [26] found very little increase in imputation accuracy by combining population- and family-based-imputation vs. population-based imputation for three dairy cattle breeds.

### Genomic prediction

The second aim of this study was to investigate the accuracy of genomic prediction based on genotypes that are imputed with different accuracies. The results revealed a small decrease in accuracy of genomic prediction based on GBLUP when the imputation accuracy was high (on average 0.95), while genomic prediction accuracy based on lowly accurate imputed 50k genotypes was lower than that based on observed 12k genotypes. This is because the correlation between genomic relationships among animals based on observed 50k genotypes and accurate imputed genotypes (Table 3) is high (0.99). Results reported for other animal species showed a similar slight decrease in genomic prediction accuracy based on accurately imputed genotypes, e.g. [10, 25, 27, 28]. Segelke et al. [27] reported a correlation of 0.98 between GEBV from observed 50k genotypes and 50k genotypes imputed from 6k/7k genotypes. The change in GEBV accuracy might not be the same when genomic prediction is based on other approaches such as Bayesian methods that rely more on the effect of individual marker alleles that are in LD with a specific QTL.

## Conclusions

We observed that imputation accuracy for purebred and crossbred animals increased as more breed-relevant haplotypes are available for the reference population. Crossbred animals required larger imputation reference sets that included genotypes for all relevant breeds. Imputation accuracy was higher when genomic relatedness between the test and reference sets increased and, depending on the availability of data, efficient imputation (faster and more accurate) is possible by selecting more informative animals for the test set. Accuracy of genomic prediction based on GBLUP did not significantly decrease compared to using actual genotypes when using accurate (>0.95) imputed genotypes, while genomic prediction based on 12k observed genotypes was more accurate than genomic prediction based on 50k genotypes that were imputed with low accuracy.
